# Teens, Tech, and Talk: Adolescents’ Use of and Emotional Reactions to Snapchat’s My AI Chatbot

**DOI:** 10.3390/bs15081037

**Published:** 2025-07-30

**Authors:** Gaëlle Vanhoffelen, Laura Vandenbosch, Lara Schreurs

**Affiliations:** 1 Media Psychology Lab, Department of Communication Sciences, Faculty of Social Sciences, KU Leuven, 3000 Leuven, Belgium; laura.vandenbosch@kuleuven.be (L.V.); lara.schreurs@kuleuven.be (L.S.); 2 Research Foundation Flanders (FWO-Vlaanderen), 1000 Brussels, Belgium

**Keywords:** artificial intelligence (AI), social media, emotional health, chatbots, adolescence, Snapchat

## Abstract

Due to technological advancements such as generative artificial intelligence (AI) and large language models, chatbots enable increasingly human-like, real-time conversations through text (e.g., OpenAI’s ChatGPT) and voice (e.g., Amazon’s Alexa). One AI chatbot that is specifically designed to meet the social-supportive needs of youth is Snapchat’s My AI. Given its increasing popularity among adolescents, the present study investigated whether adolescents’ likelihood of using My AI, as well as their positive or negative emotional experiences from interacting with the chatbot, is related to socio-demographic factors (i.e., gender, age, and socioeconomic status (SES)). A cross-sectional study was conducted among 303 adolescents (64.1% girls, 35.9% boys, 1.0% other, 0.7% preferred not to say their gender; *M_age_* = 15.89, *SD_age_* = 1.69). The findings revealed that younger adolescents were more likely to use My AI and experienced more positive emotions from these interactions than older adolescents. No significant relationships were found for gender or SES. These results highlight the potential for age to play a critical role in shaping adolescents’ engagement with AI chatbots on social media and their emotional outcomes from such interactions, underscoring the need to consider developmental factors in AI design and policy.

## 1. Introduction

About 60 years after the development of the first text-based conversational agent, ELIZA, chatbots have become widely integrated into various domains of everyday life, including education, healthcare, and entertainment (Meshram et al., 2021; Shevlin, 2024). Due to technological advancements such as generative artificial intelligence (AI) and large language models (LLMs), chatbots enable increasingly human-like, real-time conversations through text (e.g., OpenAI’s ChatGPT) and voice (e.g., Amazon’s Alexa; Schöbel et al., 2023). The resulting anthropomorphism of AI chatbots (i.e., attributing human characteristics to technology; Shevlin, 2024) seems to challenge humans to distinguish chatting with humans from chatting with chatbots. This was exemplified by “Human or Not?”, a recently developed game inspired by the Turing Test, in which 40% of the 1.5 million participants thought they were chatting with a human while this was actually an AI chatbot (Jannai et al., 2023). Consequently, the more human-like features are integrated into chatbots (e.g., expressing and understanding emotions), the more these chatbots are being used for social purposes such as romance or companionship (Shevlin, 2024). In this case, when a chatbot or conversational AI system is primarily designed to fulfill social needs and facilitate human–AI companionship, it is referred to as social AI (Brandtzaeg et al., 2025; Shevlin, 2024). Ethically, there are a lot of concerns about such social uses of AI, as they may compromise privacy, foster dependency, and blur the line between genuine and artificial relationships (e.g., Kurian, 2024).

One particular group targeted by social AI designers is youth (Brandtzaeg et al., 2025). According to recent reports, four in five (79%) UK online adolescents (aged 13–17; Ofcom, 2023), 66% of Belgian adolescents (aged 10–18; Apenstaartjaren, 2024), and 51% of US-based youth (aged 14–22; Hopelab, 2024) had used at least once a generative AI tool (e.g., ChatGPT, Snapchat’s My AI, Midjourney, and DALL-E). Next to rather utilitarian motivations to use these AI chatbots (e.g., get information, brainstorm ideas, help with schoolwork, and entertainment), adolescents use them to initiate social-supportive interactions (e.g., to discuss social and personal problems, ask advice, have casual conversations; Apenstaartjaren, 2024; Herbener & Damholdt, 2025; Hopelab, 2024). One AI chatbot that is specifically designed to meet the social-supportive needs of youth is Snapchat’s My AI.

Given the rapid integration and growing popularity of social AI chatbots in adolescents’ daily lives, there is a notable lack of research exploring which adolescents are more likely to engage with these technologies. Furthermore, it remains unclear which adolescent users are more prone to experiencing positive or negative emotions from their interactions with AI chatbots. Thus far, current literature has primarily focused on the potential of social AI to alleviate emotional and social challenges among adult users. For example, adults experiencing a lack of emotional or social companionship have reported feeling supported by chatbots such as Replika, sometimes even developing emotional attachment to these AI agents (T. Xie & Pentina, 2022). Experimental findings further show that disclosing negative emotions or venting to AI can reduce medium- to high-arousal negative affect, such as anger, frustration, and fear (Hu et al., 2025). Although research among adolescents remains limited, initial studies suggest similar patterns (e.g., Björling et al., 2019, 2021; Wiederhold, 2018), while also raising concerns about the risk of developing AI dependency, particularly among adolescents struggling with mental health issues such as anxiety or depression (Huang et al., 2024). These findings indicate that not all adolescents are equally likely to experience positive or negative emotional outcomes from interactions with social AI.

To address these research gaps, the current study aims to examine individual differences among adolescents in their use of social AI chatbots, as well as the emotional outcomes of these interactions. Specifically, the study will focus on the role of gender, age, and socioeconomic status (SES) in shaping adolescents’ engagement with the widely used My AI chatbot on Snapchat. These insights are highly needed to fuel theoretical speculations on the role of emotions in adolescents’ development of parasocial relationships with AI (e.g., Bond et al., 2025) and to provide AI literacy programs with insights on adolescents’ socio-demographic susceptibility factors to experience positive or negative interactions with social AI.

### 1.1. Adolescents and Snapchat

The My AI chatbot, powered by OpenAI’s advanced LLMs such as GPT-3.5 and GPT-4, is integrated into the Snapchat social media platform (Snapchat Support, 2024). Being used by 60% of U.S. and 94% of Belgian adolescents (Apenstaartjaren, 2024; Pew Research Center, 2023), Snapchat is one of the most popular content-sharing platforms among youth. With the aim of mimicking real-life interactions, Snapchat is characterized by affordances such as a low visibility (i.e., mainly focusing on small and well-known audiences) and a low persistence or high ephemerality (i.e., sent pictures or videos are by default only visible for a limited amount of time, ranging from a few seconds to 24 h in a Story-format; Utz et al., 2015). Bayer et al. (2016) demonstrated the low visibility in their research as they found that the platform is mainly being used for interactions with close ties. Interestingly, these interactions mostly resulted in a more positive mood. Within the context of these intimate social bubbles on Snapchat, the chatbot My AI was added to the platform in February 2023. My AI takes the form of a customizable avatar, allowing users to personalize its appearance extensively according to their preferences (Shevlin, 2024). Although this built-in chatbot only became freely accessible to all Snapchat users in April 2023, 72% of UK online adolescents had already used it by June 2023 (Ofcom, 2023). Since My AI is currently the most popular social AI tool used by adolescents and one of the first chatbots implemented in a social media platform highly popular among adolescents, the current study will focus on this specific AI chatbot.

### 1.2. User Characteristics and Perceived Outcomes

Despite the rising popularity of social AI among youth, not all adolescents’ experiences with chatbots seem to result in equally beneficial outcomes. For instance, a recent cross-sectional study among Danish high-school students showed that adolescents who engage in social-supportive conversations with chatbots such as My AI are lonelier and experience less perceived social support than non-users of chatbots (Herbener & Damholdt, 2025). Adolescents’ perceived outcomes were also studied in the Hopelab (2024) report, showing that 41% of participating youth believed generative AI would have “both a positive and negative” effect on their lives in the next decade, while 19% and 16%, respectively, thought this impact would be mostly negative or positive. Hence, individual differences seem to emerge between adolescents who use social AI or not, as well as between adolescents who experience positive or negative emotions from chatting with these bots.

Drawing upon the circular Media Practice Model, adolescents do not use media in a vacuum as their media choices and interactions are being influenced by their developing identities and socio-demographic characteristics (e.g., gender, age, and SES; Steele & Brown, 1995). In other words, adolescents’ sense of who they are or who they want to become influences their media selection, as well as their media interactions. Based upon previous research, adolescents’ gender, age, and SES might be particularly relevant factors in explaining their tendency to use social AI such as My AI and their likelihood to benefit from these interactions.

#### 1.2.1. Gender Differences

As for gender, adolescent boys and girls might not be equally prone to use My AI and flourish from these interactions. That is, several studies observed a gender bias in how generative AI is designed, pointing at a disproportional amount of AI chatbots with female characteristics such as female names or female-looking avatars (e.g., Feine et al., 2020; Unesco, 2019). One reason put forward is that female chatbots are perceived as more human-like and warmer compared to their male counterparts and are therefore more anthropomorphized and accepted as successful chatbots (Borau et al., 2021). Although girls might identify more with chatbots than boys due to their gender similarities, evidence on gender differences in individuals’ use of AI chatbots remains mixed. According to one report among US youth, boys are more likely to have ever used an AI chatbot than girls (53% vs. 48%; Hopelab, 2024), while another report (Ofcom, 2023) among UK adolescents found no gender differences when looking at AI chatbot use in general. Interestingly, the latter report also included My AI. With My AI, the gender is less stereotypical and customizable. Potentially, these features might explain why girls were slightly more likely to have used this AI chatbot than boys (54% vs. 48%).

Paradoxically, despite AI chatbots’ overall female characteristics, they are mainly designed by men and have been found to reproduce traditional gender stereotypes in their outputs (e.g., reflecting sexism and misogynism) due to biases within their LLMs (Unesco, 2024). Since these biases seem to perpetuate particularly unfavorable stereotypes about women (e.g., allocating traditional career roles to and objectification of women), girls might resonate less with the output of AI chatbots than boys, resulting in less agreeable chat interactions for them. Accordingly, girls could experience less positive emotions from interacting with My AI on Snapchat compared to boys. However, this gender difference may be specific to certain types of chatbots and might not occur in the same way with My AI. Prior research revealing the most disturbing gender biases (Unesco, 2024) focused only on open LLMs such as GPT-2, which differs from the more advanced—and potentially less biased—LLMs on which My AI is based (i.e., GPT-3.5 and GPT-4; Snapchat Support, 2024). Still, recent automated content analyses of responses generated by ChatGPT-4o-mini found persistent gender stereotypes in AI-generated ratings, stories, and images, with warmth more often attributed to portrayals of women and competence to portrayals of men (Wölfle & Schmuck, 2025). Thus, it remains unclear whether gender differences play a role in adolescents’ likeability to use and experience positive or negative emotions from interacting with My AI, specifically.

#### 1.2.2. Age Differences

Although Snapchat’s minimum age is set at 13 years old, many children younger than 13 years old already have an account on this platform. For instance, 37% of Belgian 6- to 11-year-olds are sporadically active on Snapchat (Apenstaartjaren, 2024). By the age of 18 years old, this amount even increases up to 94%. Accordingly, adolescents of all ages thus use Snapchat and have access to the My AI tool. However, it is unclear whether all adolescents actually use My AI and whether their likelihood of using it varies by age. Moreover, not all adolescents of all ages are equally digitally literate, with older adolescents being more digitally literate (Jin et al., 2020) and therefore potentially better able to regulate their emotions from chatting with them. High digital literacy likely relates to a more advanced understanding of digital media’s most beneficial uses and for which tasks they are more or less trustworthy (Vissenberg et al., 2022).

Moreover, younger and older adolescents might regulate their feelings incited by the chatbot differently. Although individuals’ general negative and positive affect levels tend to increase and decrease during adolescence, respectively (Casas & González-Carrasco, 2019), older adolescents may experience fewer negative emotions, such as anxiety, when interacting with My AI, as they are more likely to understand that it is not a real human. That is, as adolescents’ cognitive reasoning and theory of mind skills improve over time, helping them to better understand how others function, think, or feel (Blakemore, 2008), they may become better able to distinguish AI chatbots from humans. Drawing upon the dehumanization theory (Haslam, 2006), such a decrease in anthropomorphism and a shift towards perceiving AI chatbots as functional tools rather than social companions have been found to relate to a decrease in AI anxiety (Yao & Xi, 2025). However, empirical evidence regarding age differences in adolescents’ use of and emotional reactions to AI chatbots remains scarce and should therefore be explored more in-depth.

#### 1.2.3. SES Differences

Since the integration of the internet in daily life, societies are split by digital divides. Three levels of digital divides can be discerned: regarding individuals’ access to (first level), skills for (second level), and outcomes of using (third level) digital communication (Rosič et al., 2024). Individuals’ socioeconomic status (SES) is one of the most well-established factors in predicting the inequalities in adolescents’ access to digital communication (i.e., first level; Helsper, 2020). In the case of My AI, this means that adolescents with higher SES levels would be more likely to find their way to this AI chatbot than adolescents with lower SES levels. However, more recent research suggests that the role of SES has diminished in relation to this first-level digital divide (George et al., 2020). It is, consequently, unclear whether a similar dynamic will take place regarding adolescents’ SES level and their use of My AI.

Regarding the second level, differences in SES seem to impact users’ level of AI self-efficacy, which refers to the self-perceived beliefs of being capable of using AI technology (Hong, 2022). Individuals with higher SES levels seem to have more AI self-efficacy due to social and cognitive reasons (e.g., social network recommending AI products, belief of having enough experience to use these tools; Hong, 2022). Considering the third level of digital divides, SES has been found to be a less consistent predictor. While some studies identify SES as an important predictor of achieving positive but not negative outcomes after engaging with digital media (George et al., 2020; Helsper, 2020; Livingstone et al., 2021), no research has studied the role of socioeconomic inequalities in emotional outcomes after chatting with AI chatbots such as My AI.

### 1.3. The Current Study

Altogether, previous literature suggests that gender, age, and SES may play a role in the use and perceived emotional outcomes of chatting with social AI chatbots. The current study will therefore investigate whether these psychosocial factors also play a role in adolescents’ adoption and experience of chatting with more applied AI chatbots, such as Snapchat’s My AI. More precisely, a closer look will be given to adolescents’ positive (e.g., inspiration, happiness) and negative (e.g., distress and anxiety) emotions after using this AI chatbot, while controlling for general time spent on social media. This control variable is preferred over platform-specific usage (e.g., Snapchat time) to avoid conceptual and statistical overlap with the outcome variable (My AI use), increasing the risk of multicollinearity. For instance, some adolescents may use Snapchat primarily to interact with My AI, making the predictor and outcome inherently intertwined.

RQ1: Can socio-demographic characteristics (i.e., gender, age, and SES level) predict whether or not adolescents have already used My AI?
RQ2.a: Are socio-demographic characteristics (i.e., gender, age, and SES level) related to adolescents’ perceived positive emotions after using My AI?
RQ2.b: Are socio-demographic characteristics (i.e., gender, age, and SES level) related to adolescents’ perceived negative emotions after using My AI?

## 2. Materials and Methods

### 2.1. Data Collection and Sample

The current study draws on the survey data from a larger project^1^ which took place between August and September 2023, involving Belgian adolescents recruited from five secondary schools in Flanders. The study received ethical approval from the Ethics Committee of the University of Leuven, SMEC (approval number: G-2023-6631; 11 May 2023). Passive parental consent and active informed assent were obtained, and the confidentiality and anonymity of all responses were ensured. Each participant received a 5 EUR gift voucher as a token of appreciation. The hypotheses, tested model, and analytical strategy were preregistered (https://osf.io/yvzn8), and data and syntaxes (https://osf.io/w4mjh/?view_only=) can be found on the Open Science Framework (OSF).

Of the 330 participating adolescents, 27 participants were excluded from analyses due to failing or missing the attention check (*n* = 25) or reporting dubious answers (e.g., 22 years old but in the 3rd class of secondary school). This resulted in an analytical sample of 303 participants (63.0% girls, 35.3% boys, 1.0% other, 0.7% did not report their gender; *M_age_* = 15.89, *SD_age_* = 1.69). The majority was in their last year of secondary school (28%) and followed a general educational track (69.7%); 94.7% were born in [blinded] and 87.1% reported to have a West-European background. Most parents/guardians had a college or university degree (62.1% of the fathers/male guardians and 72.6% of the mothers/female guardians) and lived together (66.0%). Regarding SES, participants estimated how well off their family is in relation to others, which was overall rather high (*M_SES_* = 7.35; *SD_SES_* = 1.31; *min*: 1, *max*: 10).

### 2.2. Measures

#### 2.2.1. Socio-Demographic Variables

Participants reported their gender (1 = girl, 2 = boy, 3 = other, 99 = prefer not to say) and age (2023-birth year). The first item of the MacArthur Scale of Subjective Social Status (youth version; Goodman et al., 2001) was used to assess adolescents’ SES. Participants situated their family on a ladder with ten rungs, with the first rung (=score of 1) representing families in society who are the worst off and the tenth rung (=score of 10) representing families in society who are the best off. Higher scores indicated a higher (self-perceived) SES.

#### 2.2.2. Use of Snapchat’s My AI

To assess whether or not participants have already used Snapchat’s My AI chatbot, the following self-developed question was asked: “Did you already send something to My AI on Snapchat?” with response categories “yes” (=1) and “no” (=2). On average, 69.8% of the participants reported having already used Snapchat’s My AI.

#### 2.2.3. Emotional Reactions to Snapchat’s My AI

Participants’ emotional reactions to chatting with My AI on Snapchat were measured based upon a shortened and adapted version of the PANAS scale (Watson et al., 1988). More precisely, all participants who responded “yes” to the previous variable (i.e., use of Snapchat’s My AI) received the question “How do you feel after chatting with My AI on Snapchat?”, followed by the nine emotional reactions (positive affect: enthusiastic, interested, inspired, happy/excited; negative affect: upset, distressed, irritable, nervous, afraid). These emotions were selected out of the 20 original items based upon their applicability to an online chatbot context^2^. Answer options for each emotion ranged from never (=1) to very often (=5). Principal Axis Factoring (PAF) analysis indicated a two-factor solution with one factor comprising the positive emotional reactions (initial eigenvalue = 3.17, explained variance = 35.27%, *ω*  = 0.84, 4 items, *M* = 2.04, *SD* = 0.90) and one factor comprising the negative emotional reactions (initial eigenvalue = 2.09, explained variance = 23.27%, *ω* = 0.84, 4 items, *M* = 1.58, *SD* = 0.63).

#### 2.2.4. General Time Spent on Social Media

Following Frison and Eggermont’s (2016) approach, general time spent on social media was measured by making the distinction between a typical school day and weekend day. Participants indicated how much time they had spent on social media (e.g., Instagram, TikTok, Snapchat, BeReal, Facebook, and YouTube) but not instant messaging services (e.g., WhatsApp) in the last week. Answer options covered every half hour, ranging from 1 (=0 h, not using social media) to 49 (=24 h). Next, participants’ average daily time spent on social media was calculated by using the following formula: (time school-day × 5) + (time weekend-day × 2)/7. On average, participants spent more than 4 h a day on social media (*M* = 8.40, *SD* = 5.06).

### 2.3. Analytical Strategy

First, descriptive, exploratory factor, and reliability analyses were conducted. Afterwards, gender was dichotomized since the groups of the “other” category (*n* = 3) and the participants preferring not to mention their gender (*n* = 2) are too small to make valid group comparisons, which are needed for the main analyses (64.1% girls, 35.9% boys). Next, zero-order correlations were calculated.

For RQ1, a binary hierarchical logistic regression was conducted. Gender (with girls as reference category), age, and SES were added as predicting variables, participants’ My AI use as the dependent variable, and general time spent on social media as control variable. For RQ2.a and RQ2.b, two separate hierarchical linear regressions were run, with gender, age, and SES as predictors, and positive and negative emotional reactions, respectively, as continuous outcomes. General time spent on social media was again added as a control variable. Hence, all preregistered procedures were followed.

## 3. Results

### 3.1. Descriptives

Descriptive information and zero-order correlations between the main variables are displayed in Table 1.

### 3.2. Hypotheses

First, the logistic regression analysis to test whether gender, age, and SES are related to whether or not a participant had already used My AI or not (RQ1) showed that the model fit was good (Hosmer & Lemeshow test, *χ*^2^(8) = 9.50, *p* = 0.30) and significantly better than the model with only the control variable (*χ*^2^(3) = 15.29, *p* < 0.01, Cox and Snell *R*^2^ = 0.09, and Nagelkerke *R*^2^ = 0.13). Age was significantly and positively associated with using My AI (0 = yes, 1 = no), meaning that the odds ratio (OR) to use My AI decreased for older adolescents (OR = 1.39, *p* < 0.001, see Table 2). Gender and SES did not significantly predict My AI use.

Next, the association of gender, age, and SES with, respectively, adolescents’ perceived positive (RQ2.a) and negative emotional reactions (RQ2.b) after using My AI was tested by conducting two hierarchical linear regressions. Model fit statistics can be found in Table 3 and Table 4. Regarding RQ2.a, only age was significantly and negatively associated with experiencing positive emotions after using My AI (*β* = −0.13, *p* = 0.001). Older adolescents thus experience less positive emotions from using My AI than younger adolescents. Regarding RQ2.b, no socio-demographic variables were related to adolescents’ perceived negative emotions from using My AI.

## 4. Discussion

While AI tools such as chatbots are increasingly present in adolescents’ social lives, research on who uses them, and how they are experienced emotionally, remains limited. This gap is especially relevant given the novelty of social AI chatbots in adolescents’ daily lives. Current theoretical reflections highlight the role of novelty in shaping media experiences and the effects of emerging technologies on early adopters (Baumgartner, 2023). Popular social AI chatbots, such as My AI, are a prime example of this dynamic. Against this backdrop, our findings offer an initial look at which adolescents engage with Snapchat’s avatar-like chatbot, *My AI*, and how they emotionally respond to these interactions. Below, we reflect on the implications of these findings and discuss the study’s limitations and directions for future research.

### 4.1. Main Findings and Implications

Among the three socio-demographic factors examined, age was the only one where significant (yet small in effect size) differences emerged: Younger adolescents were more likely to chat with My AI and reported more positive experienced emotions compared to older adolescents. Although no significant links were found between age and adolescents’ negative emotional experiences with My AI in this study, a potential lack of sufficient AI literacy among younger adolescents might explain the observed significant relationships. As young adolescents are often characterized by great emotional instability (Larson et al., 2002) and underdeveloped critical thinking capacities (Dumontheil, 2014), they may be more likely to trust social chatbots as human-like conversational partners. Consequently, this may lead to biases in assessing AI-related risks, such as overestimating the positivity of their experienced emotions. For instance, when they talk about a mental health struggle (e.g., low body satisfaction), the chatbot might seem helpful (e.g., change eating pattern) but have negative consequences regarding the user’s health (e.g., eating problems). Since these chatbots can still generate unreliable answers, perpetuate stereotypical biases (e.g., related to gender), and misuse sensitive self-disclosed information, adolescents’ trust in social AI chatbots should be critically interpreted (Maeda & Quan-Haase, 2024). Enhancing AI literacy among younger adolescents through interventions could therefore help them to better understand, manage, and critically evaluate their experiences with social AI chatbots (Celik, 2023; Ng et al., 2021), potentially mitigating negative, or decreased positive, emotional impacts in the future.

Next, no significant associations were found for gender, nor for SES. The absence of significant differences between adolescents with varying SES levels might be explained by the generally high SES levels of the current sample. Moreover, the little variation in this variable might hint at a ceiling effect, which obstructs finding significant relationships. Future research should target larger and more diverse SES as well as more geographically diverse samples. The null findings regarding gender suggest that My AI’s avatar and its LLM may be more gender-neutral and less likely to perpetuate gender stereotypes compared to previous research on other chatbots and LLMs (e.g., Feine et al., 2020; Unesco, 2024). However, future research is needed to substantiate this reasoning and to explore whether any gender biases are present in My AI’s technology and whether these can be directly linked to gender differences among its users.

Overall, these results have implications for both practice and theory. First, the observed relationships between younger adolescents’ use of social AI and their experienced positive emotions may have important implications for the development of a new form of parasocial relationship. Parasocial relationships are traditionally defined as asymmetrical relationships between a media user and a media personality (e.g., influencers) that evoke a socioemotional connection (e.g., Horton & Richard Wohl, 1956). These media personalities can refer to real humans (e.g., models and celebrities) or to representations of humans (e.g., animations, holograms; Maeda & Quan-Haase, 2024). While AI chatbots fall into the latter category, Maeda and Quan-Haase (2024) suggested that the growing anthropomorphism of conversational agents, coupled with advancements in their LLMs, enables AI chatbots to foster trust and emotional connectedness, resembling human interactions. By demonstrating that especially young adolescents experience positive emotions from using My AI, our study contributes to the growing empirical support for the development of parasocial relationships between minors and (social) AI (Hoffman et al., 2021). Nevertheless, further (longitudinal) research is necessary to unravel how such parasocial relationships evolve over time.

A second implication of our findings relates to the ongoing debates surrounding the minimum age requirement for using social media platforms like Snapchat. Although many users are younger than the official age limit (Apenstaartjaren, 2024), the platform’s terms of use require users to be at least 13 years old to create an account and gain access to features like the My AI chatbot. Recently, this minimum age for social media platforms has become a contested topic in both public and policy discussions, with several governments proposing an increase in this age limit (e.g., the social media ban for under 16 years old Australian adolescents; Taylor, 2024) and asking for a more precise age verification system (e.g., Digital Services Act). Although our study specifically examines adolescents’ interactions with only one feature of Snapchat—the My AI chatbot—our results indicate that younger adolescents may sometimes benefit from the platform even more than older users. Therefore, stakeholders should consider these potential positive outcomes when shaping technology and policy, ensuring that these beneficial effects for younger adolescents are preserved in future social media designs and regulations.

### 4.2. Limitations

Despite being the first to offer empirical insights into adolescents’ socio-demographic differences in their use of and emotional responses to chatting with a social AI chatbot, some limitations should be addressed. First, since the study draws on cross-sectional data, no causal claims can be made. Although the emotional reactions variable specifically asked participants how they felt after chatting with My AI, younger adolescents are generally known to experience more positive emotions than older adolescents (Casas & González-Carrasco, 2019). This could potentially influence the emotional outcomes observed in the study. Moreover, given that this study examined only adolescents’ subjective emotional experiences, which may be prone to social desirability bias, future research should supplement self-reports with physiological measures (e.g., heart rate variability and skin conductance) or implicit assessments. Additionally, longitudinal studies are necessary to assess whether positive or negative emotions persist over time and to better understand the long-term social and mental health impacts of interacting with social AI chatbots (e.g., on identity formation, peer relationships, and self-esteem). Next, given the exclusive focus of the current study on socio-demographic factors, adolescents’ motivations for using social AI chatbots were not considered, though they may play a crucial role in the observed relationships. Drawing on uses and gratification theory (U&G), individuals’ media use and experiences are driven by their needs and whether or not these needs are satisfied, respectively (i.e., gratifications; Katz et al., 1974). Previous research applying U&G to AI-powered chatbots identified four categories of gratifications: utilitarian (e.g., information seeking), technology-based (e.g., medium appeal), hedonic (e.g., entertainment), and social (e.g., establishing and maintaining social connections; C. Xie et al., 2024). While these motivations have mostly been studied in adult samples, adolescents likely share similar motives. Recent longitudinal research also shows that emotional vulnerabilities, such as mental health problems, predict stronger social and escape motivations, which in turn increase AI dependence (Huang et al., 2024). This finding suggests that motivations, rather than AI use itself, drive emotional outcomes. Future studies should therefore examine how adolescents’ motivations and the factors that shape them contribute to their emotional responses to social AI chatbots. Socio-demographic factors such as the currently studied variables as well as additional relational factors (e.g., parental education, co-residency status, digital literacy, and prior technological experience) may indirectly influence these emotional experiences via motivational pathways. For instance, adolescents of different ages may have varying dominant motivations for using social AI chatbots, potentially explaining the age-related differences observed in their use and emotional outcomes after interacting with My AI.

Third, the current study focused exclusively on adolescents’ interactions with the My AI chatbot on Snapchat. Given its presence among users’ close ties on Snapchat, its specific LLM, and its specific affordances allowing, for instance, to customize the avatar of the chatbot, it is likely that adolescents’ interactions with other social AI chatbots on different platforms—each with distinct affordances and LLMs—may differ significantly. Since adolescents’ socio-demographic characteristics might be differently related to their use of and experiences with other chatbots, caution is needed when generalizing the findings of the current study to other social AI chatbots. Further research is therefore needed to explore how these interactions vary across different platforms.

## 5. Conclusions

In summary, the current study is the first to investigate adolescents’ use of and perceived emotional experiences with Snapchat’s My AI chatbot, highlighting the critical role of age in shaping their adoption and emotional responses to social AI chatbots. These findings not only contribute to our theoretical understanding of user–AI interactions among adolescents but also emphasize the need to consider their developmental context when designing and regulating AI chatbots. In particular, promoting AI literacy from the moment adolescents begin using social media is essential. Drawing upon social media literacy literature (Schreurs & Vandenbosch, 2021), this predominantly includes educating them that AI chatbots do not think or feel but generate responses based on large language models (cognitive literacy), and supporting emotional regulation to help manage attachment and coping with both positive and negative experiences (affective literacy). Platform developers should enhance transparency by clearly indicating AI interactions, disclosing information sources, and strengthening privacy protections in line with regulations like the Digital Services Act. These recommendations aim to foster safer and more informed adolescent engagement with social AI chatbots. Future research is advised to also study adolescents’ interactions with other social AI chatbots and delve further into their sociopsychological outcomes from these interactions.

## Figures and Tables

**Table 1:** Descriptive statistics and zero-order correlations on variables of interest.

Descriptive Statistics											
	Min.	Max.	*M*	*SD*	1	2	3	4	5	6	7
1. Gender	-	-	-	-	1						
2. SES	1	10	7.35	1.31	0.03	1					
3. Age	13	20	15.89	1.69	−0.24 ***	−0.27 ***	1				
4. Positive emotional reactions	1	5	2.04	0.8	−0.04	0.06	−0.22 **	1			
5. Negative emotional reactions	1	5	1.58	0.63	−0.09	0.05	−0.01	0.15 *	1		
6. My AI use	-	-	-	-	−0.02	0.03	0.16 **	-	-	1	
7. General time spent on social media	1	49	8.4	5.06	−0.07	−0.12 *	0.11	0.04	0.07	−0.18 **	1

Note: * *p* < 0.05, ** *p* < 0.01, *** *p* < 0.001. Gender: 1 = girl, 2 = boy. My AI use: 1 = yes, 2 = no. Since the variables of positive and negative emotional reactions were only asked to the participants who scored “1” on the variable of “My AI use”, no correlations could be conducted between these variables.

**Table 2:** Logistic regression analysis with My AI use as dependent variable and socio-demographic factors as independent variables.

	*b*	*SE*	Wald *χ*^2^	Exp(*b*)	*χ* ^2^	Cox and Snell *R*^2^	Nagelkerke’s *R*^2^
Model 1					6.79	0.04	0.06
General time spent on social media	−0.12	0.04	9.81	0.89			
Model 2					9.50	0.09	0.13
Gender	−0.08	0.30	0.07	0.07			
SES	0.06	0.11	0.36	0.36			
Age	0.33 ***	0.09	13.40	1.39			
Constant	−5.30	1.79	8.75	8.75			

Note: Gender (1 = girl, 2 = boy); My AI use (0 = yes and 1 = no); *** *p* < 0.001.

**Table 3:** Linear regression analysis with positive emotions after My AI use as dependent variables and socio-demographic factors as independent variables.

	*b*	*SE*	*β*	*t*	Adj. *R*^2^	Δ*R*^2^	F	F Change
Model 1					−0.00	0.00	0.27	0.27
General time spent on social media	0.01	0.01	0.04	0.52				
Model 2					0.04	0.06	3.02 *	3.93
General time spent on social media	0.01	0.01	0.06	0.08				
Gender	−0.17	0.13	−0.09	−1.24				
SES	0.00	0.05	0.01	0.08				
Age	−0.13	0.04	−0.24 **	−3.26				

Note: Gender (1 = girl, 2 = boy); * *p* < 0.05, ** *p* < 0.01.

**Table 4:** Linear regression analysis with negative emotions after My AI use as dependent variables and socio-demographic factors as independent variables.

	*b*	*SE*	*β*	*t*	Adj. *R*^2^	Δ*R*^2^	F	F Change
Model 1					0.00	0.01	1.03	1.03
General time spent on social media	0.01	0.09	0.07	1.01				
Model 2					−0.01	0.01	0.75	0.66
General time spent on social media	0.01	0.01	0.07	1.03				
Gender	−0.11	0.10	−0.09	−1.17				
SES	0.03	0.04	0.05	0.70				
Age	−0.01	0.03	−0.02	−0.28				

Note: Gender (1 = girl, 2 = boy).

## Data Availability

The data that support the findings of this study are openly available in OSF at https://osf.io/w4mjh/?view_only=.

## Notes

1 Only one other study draws upon the same sample, yet has completely different research objectives (see its preregistration on OSF: https://osf.io/yvzn8) and has no overlapping main variables.

2 The dropped items for negative affect were guilty, scared, hostile, ashamed, and jittery; for positive affect: strong, proud, alert, determined, attentive, and active.

## Abbreviations

The following abbreviations are used in this manuscript:

AI	Artificial Intelligence
LLMs	Large Language Models
SES	Socioeconomic Status
OSF	Open Science Framework
